# Elucidating the Diversity and Potential Function of Nonribosomal Peptide and Polyketide Biosynthetic Gene Clusters in the Root Microbiome

**DOI:** 10.1128/mSystems.00866-20

**Published:** 2020-12-22

**Authors:** Barak Dror, Zongqiang Wang, Sean F. Brady, Edouard Jurkevitch, Eddie Cytryn

**Affiliations:** a Institute of Soil, Water and Environmental Sciences, Agricultural Research Organization, Rishon-Lezion, Israel; b Department of Plant Pathology and Microbiology, The Robert H. Smith Faculty of Agriculture, Food and Environment, The Hebrew University of Jerusalem, Rehovot, Israel; c Laboratory of Genetically Encoded Small Molecules, The Rockefeller University, New York, New York, USA; Wageningen University

**Keywords:** soil microbiome, root microbiome, polyketides, nonribosomal peptides, secondary metabolites, plant-microbe interactions, secondary metabolism

## Abstract

Polyketides (PKs) and nonribosomal peptides (NRPs) are two microbial secondary metabolite (SM) families known for their variety of functions, including antimicrobials, siderophores, and others. Despite their involvement in bacterium-bacterium and bacterium-plant interactions, root-associated SMs are largely unexplored due to the limited cultivability of bacteria. Here, we analyzed the diversity and expression of SM-encoding biosynthetic gene clusters (BGCs) in root microbiomes by culture-independent amplicon sequencing, shotgun metagenomics, and metatranscriptomics. Roots (tomato and lettuce) harbored distinct compositions of nonribosomal peptide synthetases (NRPSs) and polyketide synthases (PKSs) relative to the adjacent bulk soil, and specific BGC markers were both enriched and highly expressed in the root microbiomes. While several of the highly abundant and expressed sequences were remotely associated with known BGCs, the low similarity to characterized genes suggests their potential novelty. Low-similarity genes were screened against a large set of soil-derived cosmid libraries, from which five whole BGCs of unknown function were retrieved. Three clusters were taxonomically affiliated with *Actinobacteria*, while the remaining were not associated with known bacteria. One *Streptomyces*-derived BGC was predicted to encode a polyene with potential antifungal activity, while the others were too novel to predict chemical structure. Screening against a suite of metagenomic data sets revealed higher abundances of retrieved clusters in roots and soil samples. In contrast, they were almost completely absent in aquatic and gut environments, supporting the notion that they might play an important role in root ecosystems. Overall, our results indicate that root microbiomes harbor a specific assemblage of undiscovered SMs.

**IMPORTANCE** We identified distinct secondary-metabolite-encoding genes that are enriched (relative to adjacent bulk soil) and expressed in root ecosystems yet almost completely absent in human gut and aquatic environments. Several of the genes were distantly related to genes encoding antimicrobials and siderophores, and their high sequence variability relative to known sequences suggests that they may encode novel metabolites and may have unique ecological functions. This study demonstrates that plant roots harbor a diverse array of unique secondary-metabolite-encoding genes that are highly enriched and expressed in the root ecosystem. The secondary metabolites encoded by these genes might assist the bacteria that produce them in colonization and persistence in the root environment. To explore this hypothesis, future investigations should assess their potential role in interbacterial and bacterium-plant interactions.

## Abstract (precis)

We identified distinct secondary-metabolite-encoding genes that are enriched (relative to adjacent bulk soil) and expressed in root ecosystems yet almost completely absent in human gut and aquatic environments. Several of the genes were distantly related to genes encoding antimicrobials and siderophores, and their high sequence variability relative to known sequences suggests that they may encode novel metabolites and may have unique ecological functions.

## INTRODUCTION

Soil is an extremely diverse ecosystem that contains a myriad of micro- and macroorganisms, including nematodes, arthropods, fungi, and bacteria. The rhizosphere is a narrow region of soil directly influenced by root exudates and mucilage (1, 2). This “hot spot” of organic matter and nutrients “enriches” a specific fraction of the soil microbial community known as the root microbiome, which is significantly different than the surrounding soil microbiome (3). Over the past 2 decades, several studies have linked specific constituents of the root microbiome to enhanced plant growth and development and inhibition of soilborne plant pathogens (4) by direct antagonism and/or induced systemic resistance (5). These functions are often facilitated by the vast array of secondary metabolites (SMs) produced by root-associated bacteria, which play a key role in inter- and intraspecies interactions (6, 7).

Many important soil and root-associated bacterial SMs are nonribosomal peptides (NRPs) or polyketides (PKs), produced by nonribosomal peptide synthetases (NRPSs) or polyketide synthases (PKSs), respectively. These are encoded on large biosynthetic gene clusters (BGCs) that often exceed 50,000 bp (8). Enzymatic complexes in these families follow a similar biosynthetic logic wherein molecules are assembled in an iterative building process using conserved domains that are organized in modules (9, 10). NRPSs and PKSs are responsible for the synthesis of a wide array of siderophores, toxins, pigments, and antimicrobial compounds (11) that are believed to play pivotal roles in bacterial adaptation to soil and rhizosphere ecosystems and in plant health and development (12). Despite their ecological (rhizosphere competence) and translational (biocontrol agents and novel antimicrobial compounds for plant protection) importance, little is known about the occurrence, diversity, and dynamics of NRPSs and PKSs in root ecosystems or their role in intra- and intermicrobial and plant-bacterium interactions.

A major challenge in exploring the role and function of SMs in soil stems from the fact that the majority of bacteria cannot be cultivated using conventional methods, making it difficult to study these bacteria and the diversity, expression, and function of the metabolites that they produce (13). Despite the progress made in culturing techniques, our capacity to isolate soil and root-associated bacteria is highly constrained, primarily because it is challenging to mimic the natural conditions required for growing these bacteria (14). Furthermore, many bacterial BGCs are silent under laboratory conditions, and therefore, the metabolites that they encode are extremely challenging to isolate (15).

To circumvent the above-mentioned barriers, a myriad of culture-independent sequencing-based and omics tools have been developed to reveal the scope and composition of soil-derived BGCs encoding NRPSs and PKSs (16, 17) and to infer the chemical composition and structure of the metabolites produced by these synthases (18, 19). For instance, amplicon sequencing-based approaches have been developed to target short fragments within adenylation (AD) (in NRPS) and ketosynthase (KS) (in PKS) domains. These amplicons can be used to ascertain the diversity and abundance of bacterial BGCs in complex environments as both AD and KS domains are important (in concert with other components) for the assembly, and thus the identity and activity, of the synthesized metabolites (20, 21). To date, a few studies have explored the diversity and composition of bacterial SM-encoding BGCs in soil, demonstrating the vast genetic diversity and novelty of NRPS and PKS genes (22, 23). However, little is known regarding the distribution of these gene families in the root microbiome, and their functional role in this complex community remains an enigma (24).

This study proposes a unique approach to analyze the diversity and potential functions of NRPSs and PKSs in the root, specifically focused on elucidating (i) the composition and diversity of NRPS- and PKS-encoding genes in the root environment relative to adjacent bulk soil, (ii) NRPS and PKS composition and expression in the root as a function of plant type, (iii) the sequence and inferred SM structures of whole bacterial BGCs that are highly abundant or expressed in root environments, and (iv) the occurrence of root-enriched bacterial BGCs in other ecosystems.

## RESULTS

### Composition and diversity of NRPS and PKS genes in roots versus bulk soil.

To determine the composition and diversity of NRPSs and PKSs in tomato and lettuce root samples relative to bulk soil (previous studies targeting this controlled lysimeter system showed that bulk soils from tomato and lettuce microbiomes were almost identical, and therefore, only tomato soil was analyzed here), we applied a previously described amplicon sequencing approach to amplify the conserved adenylation (AD) and ketosynthase (KS) domains of NRPSs and PKSs, respectively (23). Overall, sequencing yielded totals of 1,850,442 and 2,174,020 raw KS and AD reads with average read lengths of 280 bp and 235 bp, respectively (see Table S1 in the supplemental material). Further filtering steps using QIIME2 and DADA2 denoising methods resulted in 2,980 and 3,269 nonredundant KS and AD domain sequences, respectively.

10.1128/mSystems.00866-20.7 TABLE S1 Amplicon sequencing processing information. Download Table S1, CSV file, 0.01 MB. Copyright © 2020 Dror et al. 2020 Dror et al. This content is distributed under the terms of the Creative Commons Attribution 4.0 International license.

**FIG 1:**
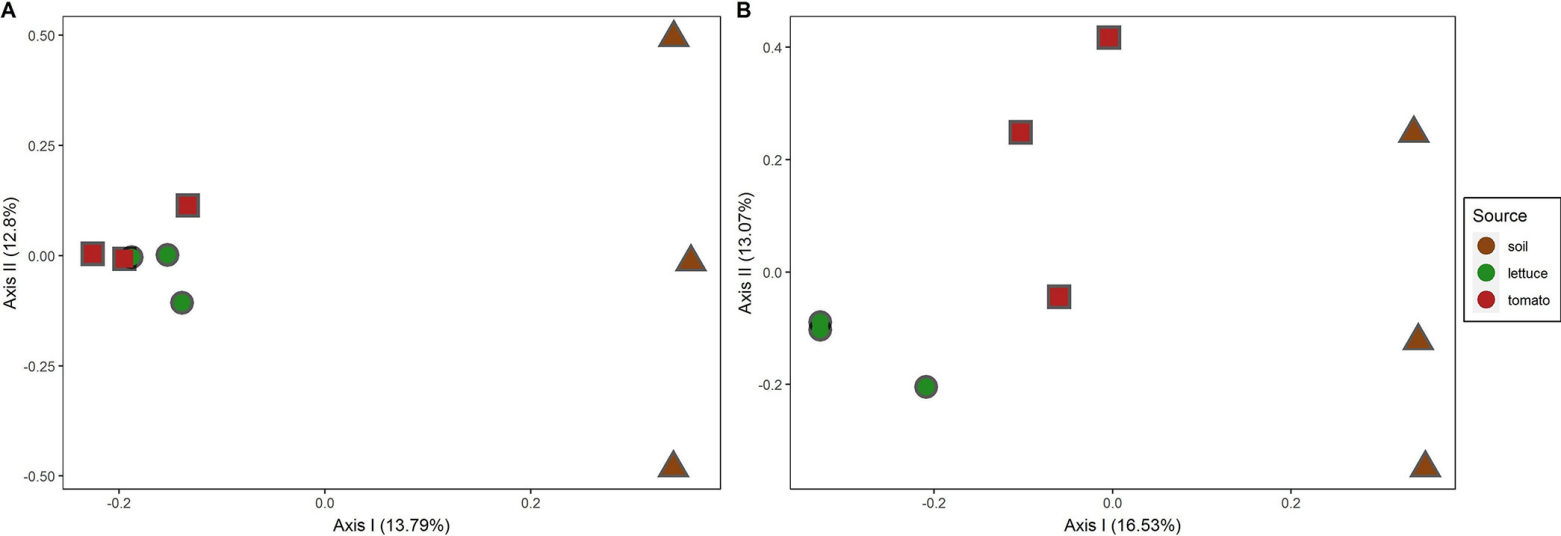
Principal-coordinate analysis (PCoA) of lettuce root, tomato root, and soil (from tomato lysimeters) samples using the Bray-Curtis similarity index. (A) Analysis of KS (from PKS) domain amplicons (roots versus soil ANOSIM statistic *R* = 0.332 [*P* < 0.05]; tomato versus lettuce versus soil ANOSIM *R* = 0.432 [not significant {NS}]). (B) Analysis of AD (NRPS) domain amplicons (roots versus soil ANOSIM statistic *R* = 0.308 [*P* < 0.01]; tomato versus lettuce versus soil ANOSIM *R* = 0.6872 [*P* < 0.01]).

We observed significantly greater diversity of both AD and KS domains in the bulk tomato soil than in the adjacent roots (Fig. S1A and C). In contrast, no difference in diversity was observed between tomato and lettuce roots for either of the SM-encoding domains (Fig. S1B and D). To assess differences in AD and KS domain diversity between samples, a principal-coordinate analysis (PCoA) and analysis of similarity (ANOSIM) using the Bray-Curtis similarity index were performed (Fig. 1A and B). The KS and AD domain profiles of the roots (from both tomato and lettuce) formed distinct clusters, which were significantly different from those of the adjacent bulk soil (*R* = 0.332 [*P* < 0.05] for PKS; *R* = 0.308 [*P* < 0.01] for NRPS).

10.1128/mSystems.00866-20.1 FIG S1 Alpha diversity (1-Simpson similarity index) of soil and root (tomato and lettuce) samples. (A and B) AD sequences (NRPS); (C and D) KS sequences (PKS). Asterisks represent statistical significance between samples (by a Tukey honestly significant difference [HSD] test) (*, *P* < 0.001; **, *P* < 0.0001; NS, not significant). Download FIG S1, TIF file, 0.1 MB. Copyright © 2020 Dror et al. 2020 Dror et al. This content is distributed under the terms of the Creative Commons Attribution 4.0 International license.

To explore the potential novelty of root-associated SM-encoding genes, the amplified AD and KS domain sequences from the root and soil samples were first aligned against the MIBiG (Minimum Information about a Biosynthetic Gene Cluster) repository (25) using blastp, with a >50% amino acid sequence identity cutoff, and then grouped according to their identity to the MIBiG reference genes (Table 1). On average, more than 25% of the AD and almost 13% of the KS domain sequences in the root environment showed less than 50% amino acid identity with genes found in the MIBiG database (characterized as “unassigned”), whereas fewer than 1% and 6% of the AD and KS sequences, respectively, shared over 85% similarity to the reference MIBiG genes. These results demonstrate the profusion of potentially novel SM-encoding genes in both root and soil environments.

**TABLE 1:** Abundance of NRPS (AD) and PKS (KS) amplicons based on levels of similarity to MIBiG NRPS and PKS reference genes in the tomato and cucumber root and tomato soil samples^*a*^

Gene	Identity (%)	% of sequences (total no. of hits)			
		Tomato	Lettuce	Avg (roots)	Soil
AD	Unassigned	24.1 (225)	28.6 (202)	26.10 (427)	14.0 (234)
	50–70	56.2 (523)	53.9 (381)	55.2 (904)	66.8 (1,114)
	70–85	18.7 (174)	16.2 (115)	17.6 (289)	18.3 (305)
	85–95	0.8 (8)	1.1 (8)	0.97 (16)	0.72 (12)
	95–100	0.0	0.0	0.0	0.06 (1)

KS	Unassigned	10.2 (108)	14.4 (202)	12.6 (310)	5.0 (17)
	50–70	46.2 (486)	46.5 (652)	46.4 (1,138)	56.2 (190)
	70–85	37.5 (394)	32.1 (451)	34.4 (845)	33.7 (114)
	85–95	5.1 (54)	6.0 (85)	5.6 (139)	4.7 (16)
	95–100	0.7 (8)	0.7 (11)	0.7 (19)	0.2 (1)

a Identity groups were determined as unassigned (<50% amino acid sequence identity) and 50 to 70%, 70 to 85%, 85 to 95%, and 95 to 100% sequence identity. Numbers outside parentheses indicate the percentages of sequences associated with each group, and numbers inside parentheses indicate the total number of hits for each group.

### Pinpointing predicted root-enriched NRPs and PKs.

As SMs are known to play critical roles in bacterium-bacterium and bacterium-plant interactions, we were interested in the associated metabolites synthesized by BGCs whose AD or KS domains were highly abundant and enriched in tomato or lettuce roots relative to bulk soil. To do so, the MIBiG-aligned amplicons were annotated to the corresponding BGC-associated metabolites, using a cutoff E value of <10^−40^. Sequences that did not meet these criteria were defined as “unknown.” Previous analyses have shown that compared to reference KS or AD domain sequences, amplicons with E values of <10^−40^ are likely derived from the same BGC family as the reference sequence and thus may be inferred to encode a similar function (26–29).

**FIG 2:**
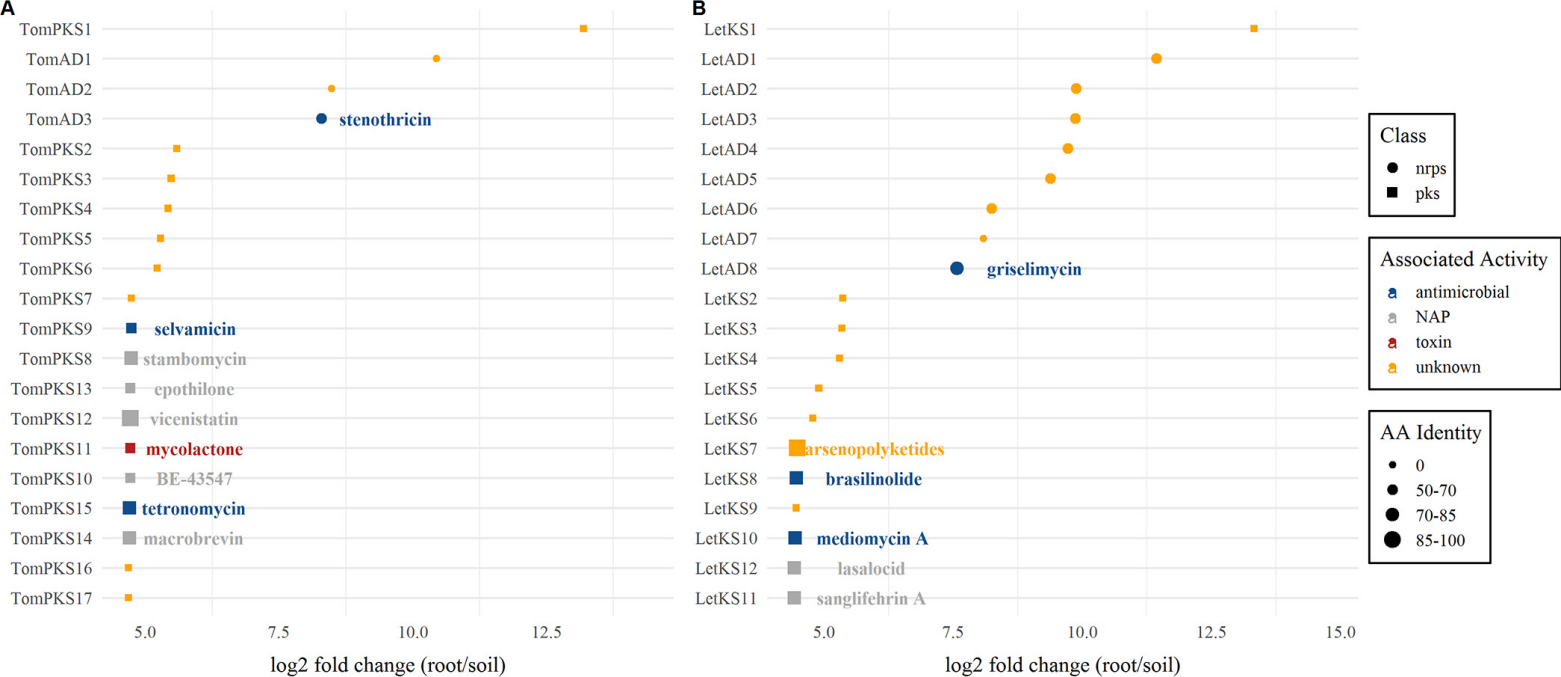
Differential abundances of the top 20 highly abundant NRPS and PKS amplicons in tomato (A) and lettuce (B) root microbial communities relative to bulk tomato soil. DESeq2 was used to calculate log_2_ fold changes and test for significance (adjusted *P* value [false discovery rate {FDR}] of <0.1). Associated metabolites (based on the MIBiG repository) are presented only for sequences with E values of <10^−40^ and >50% amino acid (AA) identity. Identity bars represent amino acid identity to the MIBiG reference sequence (0, not identified). NAP, non-antimicrobial pharmaceutical (e.g. anti-tumor, anti-inflammatory, etc.).

A differential abundance analysis using DESeq2 of the top 20 highly abundant AD and KS amplicons revealed that 55% (11/20) and 70% (14/20) of the amplicons in both of the plant root samples (tomato and lettuce, respectively) (adjusted *P* value of <0.1; log_2_ fold change of >5) were not associated with known BGCs; thus, their associated metabolites cannot be inferred (Fig. 2). Nonetheless, several root-enriched AD and KS domain sequences (9 in tomato and 6 in lettuce) were above these threshold values and thus can be considered congeners to known metabolites. These included the nonribosomal peptides stenothricin (30) and griselimycin (31), whose BGC NRPS analogues were highly abundant in the tomato and lettuce roots, respectively, and were less profuse in the bulk soil. While we cannot determine the actual role of the metabolites potentially encoded by these enriched BGCs, both stenothricin and griselimycin are known for their antimicrobial activity.

**FIG 3:**
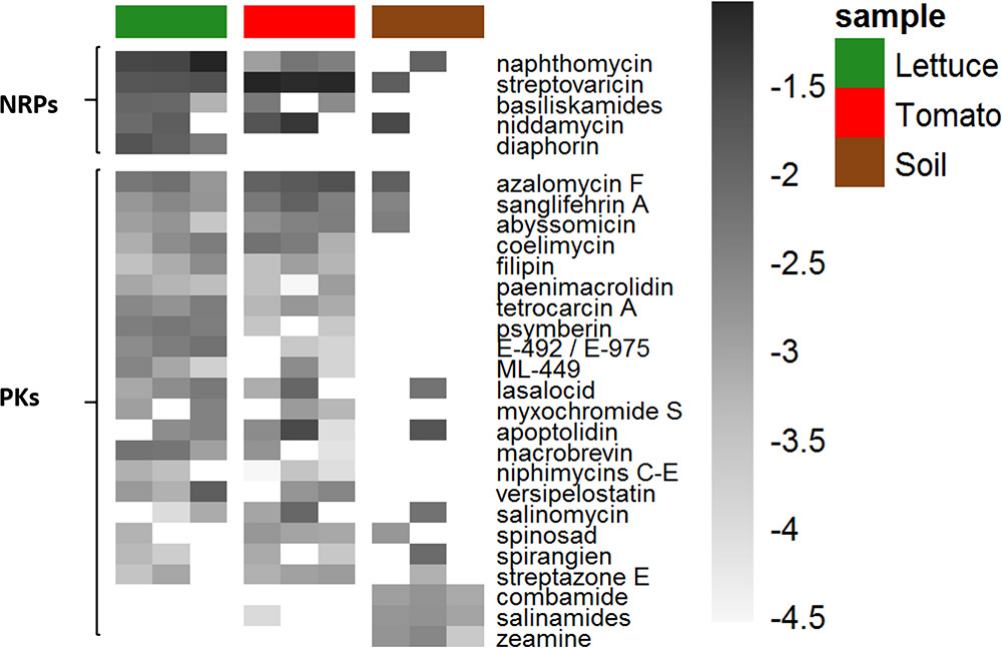
Relative abundances of NRPS (top)- and PKS (bottom)-associated metabolites based on MIBiG annotations. Only AD and KS amplicon hits to reference MIBiG sequences with over >50% amino acid identity and an E value of <10^−40^ were included. Counts were normalized and log_10_ transformed. Only associated metabolites that were present in >2 different samples are shown.

Next, we calculated the relative abundances of amplicons that were associated with known metabolites (based on the criteria described above) in the different root and soil samples (Fig. S2). To pinpoint associated BGCs that may play a role in adaptation to the root environment, we focused our analysis on inferred metabolites that were present in at least four of the root samples (tomato and lettuce) and in no more than one soil sample (Fig. 3). In addition, to identify BGCs specifically relevant to soil, we also selected inferred metabolites that were present in all three soil samples and in no more than one root-associated sample. For NRPs, we found BGCs associated with four metabolites that were highly abundant in both of the root samples (e.g., the *Streptomyces*-derived antibiotic macrolide family streptovaricin). For PKs, we again found several highly abundant *Streptomyces*-derived inferred metabolites, among others. These included lasalocid, sanglifehrin A, and azalomycin A. Interestingly, amplicons associated with the two former metabolites were also found to be highly enriched in roots relative to soil for lettuce (Fig. 2B). Overall, we found that 26 associated metabolites were present in at least one of the root-associated samples and completely absent in the soil samples, e.g., diaphorin (in lettuce) and basiliskamides (present in both tomato and lettuce) (Fig. S2).

10.1128/mSystems.00866-20.2 FIG S2 Relative abundances of NRPS (A)- and PKS (B)-associated metabolites based on MIBiG annotations. Only AD and KS amplicon hits to reference MIBiG sequences with >50% amino acid identity and an E value of <10^−40^ were included. Counts were normalized and log_10_ transformed. Only associated metabolites that were present in >2 different samples are shown. Download FIG S2, TIF file, 1.3 MB. Copyright © 2020 Dror et al. 2020 Dror et al. This content is distributed under the terms of the Creative Commons Attribution 4.0 International license.

Due to the potential biases associated with the above-described PCR-based approach, we analyzed previously reported (32) shotgun metagenomes of the same tomato and lettuce root samples (*n* = 3 each). Assembled open reading frame (ORF) sequences from the metagenomes identified using Prodigal were aligned against the MIBiG database, generating a list of the 50 most abundant NRPSs and PKSs in each of the root data sets (representing the normalized abundance within samples by plotting the coefficient of variance [CV] for each gene) (Fig. S3A and B). In addition, as gene clusters are often silent or expressed under very specific conditions, we evaluated gene expression in parallel to gene occurrence to uncover active BGCs with ecological importance in the highly dynamic root ecosystem. Thus, in parallel to the shotgun metagenome analysis described above, we applied a similar analytical approach using the previously collected shotgun metatranscriptomes (32) to identify NRPSs and PKSs with enhanced expression in lettuce and/or tomato root microbiomes. Interestingly, 60% (30/50) and 46% (23/50) of the AD and KS domains that were highly abundant in the tomato and lettuce root samples, respectively, were highly expressed as well (Fig. S3).

10.1128/mSystems.00866-20.3 FIG S3 The 50 most abundant NRPSs and PKSs in tomato (A) and lettuce (B) root microbiomes extrapolated from shotgun metagenomic data. Relative abundance represents the mean from three repeats per sample. The CV (coefficient of variance) was determined as the standard deviation (STD)/mean per sequence. Green-circled points represent sequences that are highly abundant in the corresponding metatranscriptome data sets per sample type, indicating that these are also highly expressed. Download FIG S3, TIF file, 1.3 MB. Copyright © 2020 Dror et al. 2020 Dror et al. This content is distributed under the terms of the Creative Commons Attribution 4.0 International license.

**FIG 4:**
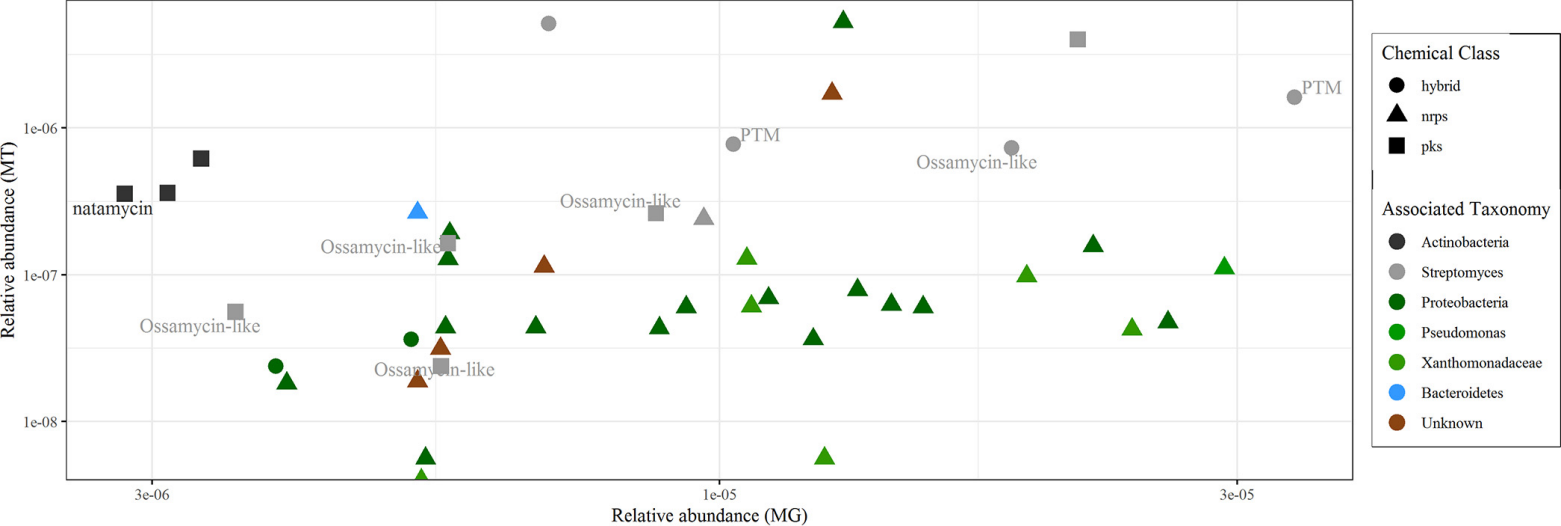
Relative abundances and taxonomy of highly abundant NRPSs/PKSs in tomato and lettuce root metagenomes (MG) and metatranscriptomes (MT). Shapes represent BGC-associated chemical classes. Taxonomy was assigned using MEGAN 5.10 and the lowest-common-ancestor (LCA) classification algorithm. Only low-CV sequences were chosen.

Next, we filtered the highly abundant hits based on their CV values (<50) in order to analyze sequences with lower dispersion levels within tomato and lettuce root-associated samples, followed by taxonomic annotation using MEGAN. The resulting 42 sequences were clustered into two main phyla: *Actinobacteria* (13/42) and *Proteobacteria* (25/42). Several sequences could not be assigned a taxonomic affiliation, and one was assigned to the *Bacteriodetes* phylum (Fig. 4). While we could not infer the associated metabolites synthesized by most of these highly abundant sequences (including all of those assigned to the phylum *Proteobacteria*), suggesting their potential novelty, we managed to annotate several of the *Actinobacteria*-associated BGCs. These were distantly associated with ossamycin (5 hits) and polycyclic tetramate macrolactams (PTMs) (2 hits, including the most highly abundant NRPS/PKS-related sequence and the 5th most expressed). Ossamycin is a known antifungal and cytotoxic macrocyclic polyketide originally isolated from soil-associated Streptomyces hygroscopicus subsp. *ossamyceticus* (33, 34), while PTMs are a family of biologically important metabolites, including HSAF (heat-stable antifungal factor), ikarugamycin, and clifednamides, generally associated with different isolates of *Actinobacteria* and *Gammaproteobacteria* (35).

Finally, to evaluate the extent to which the amplicon sequencing method was able to detect NRPSs and PKSs in the targeted samples relative to the PCR-independent shotgun metagenomic analyses, we analyzed the distribution of total MIBiG-associated genes in all four data sets (lettuce and tomato NRPS and PKS amplicons and tomato and lettuce metagenomes) (Fig. S4). In general, approximately 34% and 25% of the MIBiG-characterized genes were found in both amplicon sequences and metagenomes of the lettuce and tomato roots, respectively. In contrast, approximately 33% and 20% of the tomato and lettuce genes, respectively, were detected only in the shotgun metagenomic data sets. Fewer than 4% and 5% of the NRPSs and PKSs were found only within the tomato and lettuce amplicon sequencing data sets (28 and 35 genes in tomato and lettuce, respectively), and fewer than one-fifth (139 genes or 17.2%) were common to all four culture-independent data sets.

10.1128/mSystems.00866-20.4 FIG S4 Mined NRPSs and PKSs from amplified AD and KS domains in tomato and lettuce roots, tomato metagenomes, and lettuce metagenomes. AS, amplicon sequencing; MG, metagenome. Numbers in circles represent the number of unique MIBiG-derived genes found within each data set, while numbers in parentheses represent the percentage of unique genes from the total. Download FIG S4, TIF file, 0.2 MB. Copyright © 2020 Dror et al. 2020 Dror et al. This content is distributed under the terms of the Creative Commons Attribution 4.0 International license.

### Extraction and environmental distribution of whole SM-encoding gene clusters.

**FIG 5:**
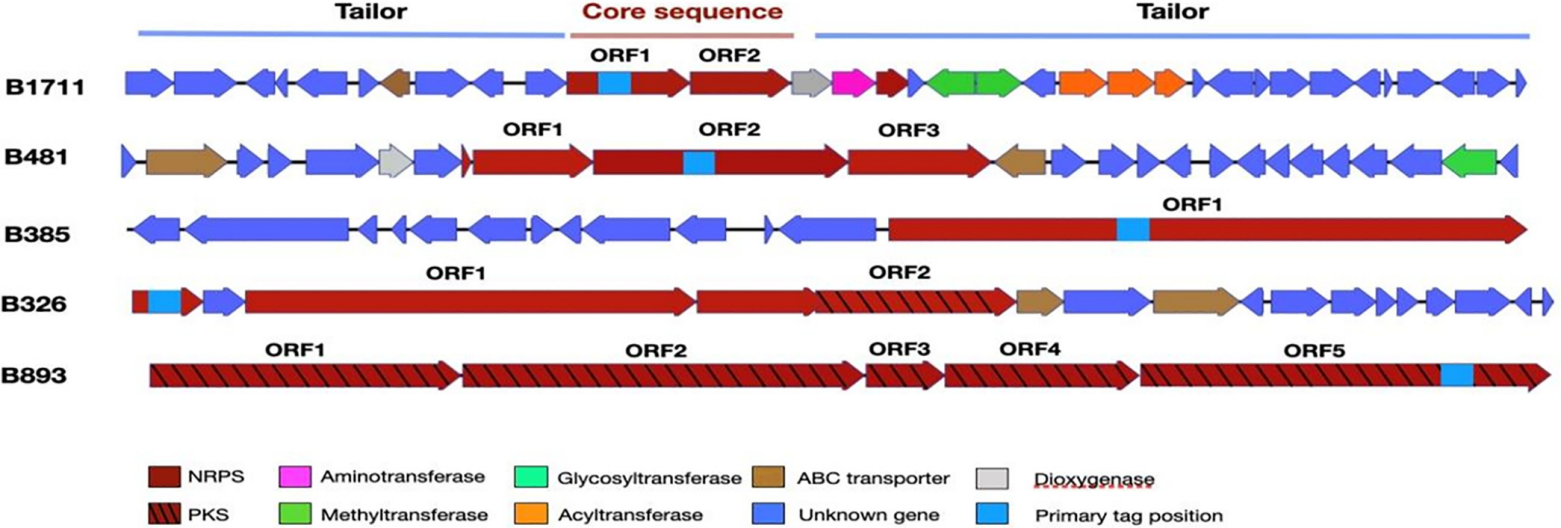
Overview of retrieved (from archived soil cosmid libraries) biosynthetic gene clusters containing AD and KS domain contigs that were abundant and/or highly expressed in tomato and cucumber root microbiomes.

**FIG 6:**
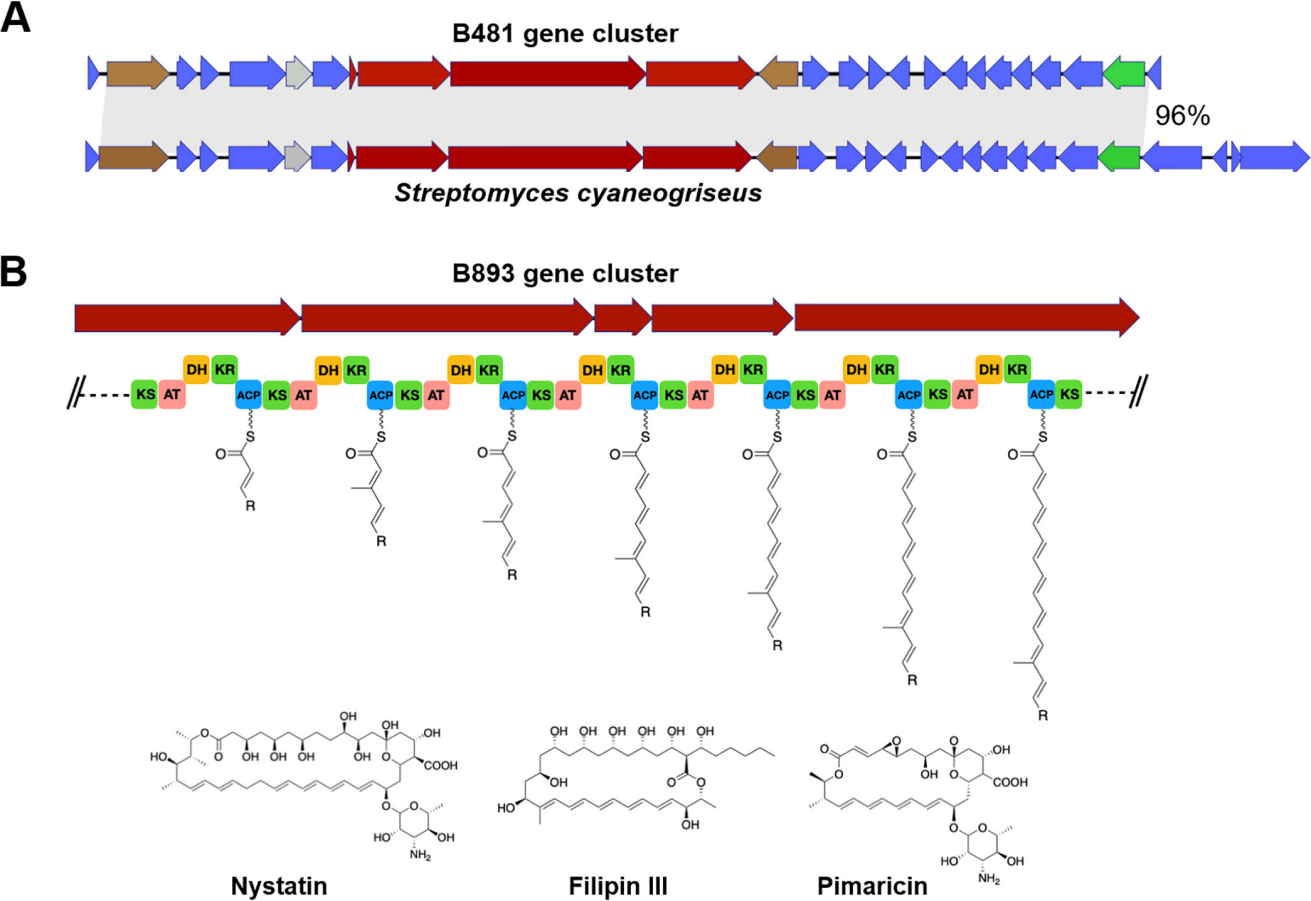
(A) Comparison of the B481 cluster and the related BGC in S. cyaneogriseus. The gray area indicates the aligned region. (B) Structure prediction for PKS modules present on clone B893. Examples of known polyene antifungal agents are shown at the bottom. KS, ketosynthase; AT, acyltransferase; DH, dehydratase; KR, ketoreductase; ACP, acyl carrier protein.

The identification of NRPSs and PKSs that were either enriched in roots relative to adjacent bulk soil or abundant and/or highly expressed in lettuce and/or tomato roots encouraged us to capture whole BGCs associated with these sequences in order to shed light on their phylogenetic affiliation and potentially infer their function and chemical structure. This was achieved by screening the root-associated NRPS and PKS candidate sequences identified in this study against a large set of previously collated soil and rhizosphere cosmid libraries using the bioinformatic platform eSNaPD (29) (see Materials and Methods for the full pipeline). Five cosmid library targets showed low E values and high nucleotide sequence identities (>75%) to candidate NRPSs or PKSs. Sequencing and annotation of the metagenomic insert captured in each cosmid revealed three NRPS and two hybrid NRPS/PKS gene clusters (Fig. 5). Based on gene content and sequence identity, the identified gene clusters were not identical to any BGCs associated with known metabolites. The NRPS and PKS ORFs of two recovered clones (B326 and B385) were not affiliated with any known bacterial taxa (<50% nucleic acid identity to the NCBI database), while the other three clones were related to genes from *Actinobacteria* (Table 2). Of the cosmids recovered from the metagenomic libraries, clone B481 was nearly identical to an uncharacterized NRPS BGC found in the genome of Streptomyces cyaneogriseus (Fig. 6A). The only predicted chemical structure that we could infer from the recovered BGCs was for clone B893, which was related to an uncharacterized PKS gene cluster found in the genome of Saccharothrix saharensis, a filamentous actinobacterium isolated from desert soil. A detailed bioinformatic analysis of its PKS domains revealed that the gene cluster likely encodes an extended polyene substructure (Fig. 6B). The seven PKS modules captured on the clone all contain dehydratase (DH) and ketoreduction (KR) domains, indicating that each module introduces a double bond into the polyketide backbone (Fig. 6B). While polyene substructures like this are seen in a number of natural products (36, 37), they are most commonly seen in polyene antifungal agents, including many that are derived from *Streptomyces* species (e.g., cyphomycin, nystatin, filipin, and pimaricin). This may suggest that the BGC encodes an antifungal compound.

**TABLE 2:** Predicted taxonomy and source data set of recovered cosmids^*a*^

Gene cluster	Domain (metabolite)	Coverage (%)	Identity (%)	GenBank accession no.	Source data set	Affiliated organism	Taxon
1711	B1711-1 (NRPS)	84	58.92	WP_136726459.1	Lettuce	*Streptomyces* sp. NEAU-C40	*Actinobacteria*
	B1711-2 (NRPS)	97	69.36	WP_136726460.1		*Streptomyces* sp. NEAU-C40	*Actinobacteria*

481	B481-1 (NRPS)	99	92.97	WP_052808563.1	Tomato	*Streptomyces cyaneogriseus*	*Actinobacteria*
	B481-2 (NRPS)	99	96.60	WP_052808562.1		*Streptomyces cyaneogriseus*	*Actinobacteria*
	B481-3 (NRPS)	99	93.30	WP_044379030.1		*Streptomyces cyaneogriseus*	*Actinobacteria*

385	B385 (NRPS)	UA	UA		Lettuce	UA	

326	B326-1 (NRPS)	UA	UA		Lettuce	UA	
	B326-2 (PKS)	UA	UA			UA	

893	B893-1 (PKS)	98	90.90	WP_141974662.1	Tomato	*Saccharothrix saharensis*	*Actinobacteria*
	B893-2 (PKS)	99	89.02	WP_141974663.1		*Saccharothrix saharensis*	*Actinobacteria*
	B893-3 (PKS)	98	92.31	TQM77832.1		*Saccharothrix saharensis*	*Actinobacteria*
	B893-4 (PKS)	100	87.62	WP_141974664.1		*Saccharothrix saharensis*	*Actinobacteria*
	B893-5 (PKS)	97	89.78	WP_141974665.1		*Saccharothrix saharensis*	*Actinobacteria*

a UA, unassigned. Only affiliated organisms with >50% identity are shown.

**FIG 7:**
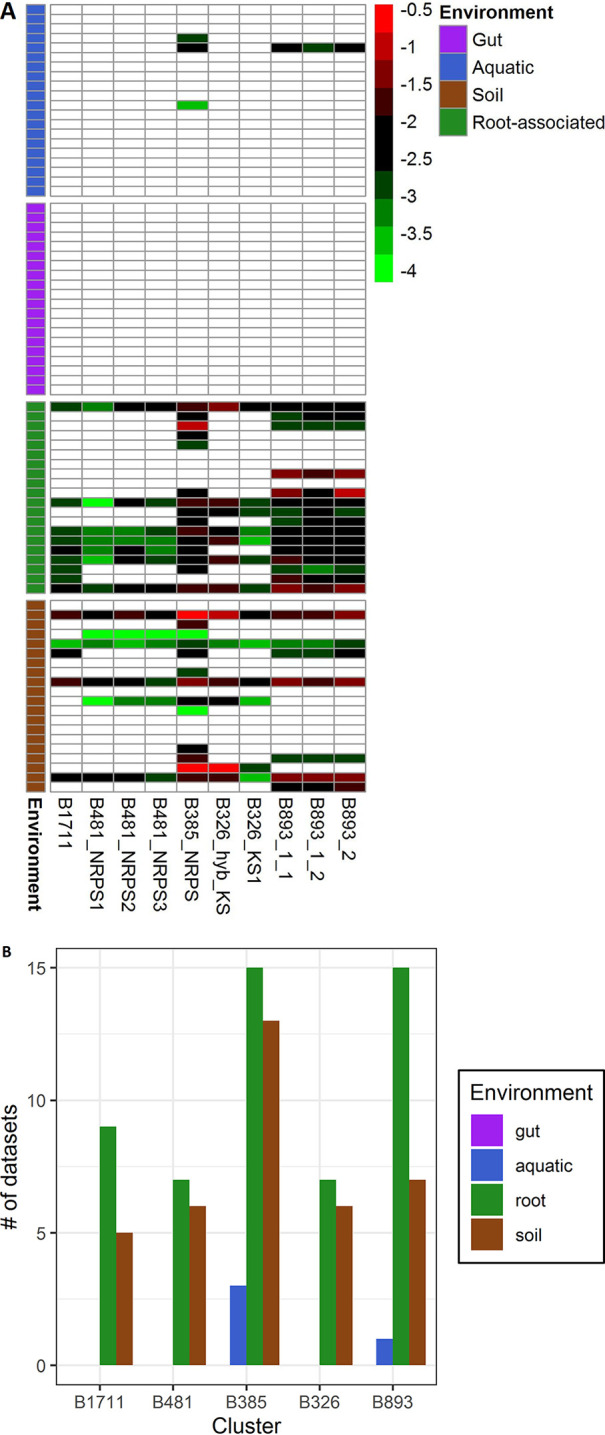
(A) Relative abundances of clone-recovered AD and KS domains in shotgun metagenomes (*n* = 20) from 4 environmental ecosystems: gut (animal and human), aquatic (freshwater and marine), soil, and root associated. Analysis was conducted using the IMG Blastn feature with an E value of <10^−5^ and an identity of >85%. Counts were normalized with *rpoB* and *gyrB* for each data set. (B) Presence/absence of each recovered gene cluster in the different environments. Only gene clusters where all AD/KS domains were found are considered present.

The five recovered BGCs were initially targeted due to their abundance in tomato and lettuce root data sets, suggesting a link to root ecosystems. To test this hypothesis, we assessed the abundances of the five BGCs in a large collection of publicly available shotgun metagenomes (20 metagenomes from each environment) from four distinct environments, targeting gut (animal and human), aquatic (freshwater and marine), soil (different soil types), and root-associated (various plant species) data sets. Our analysis demonstrated that the recovered BGCs are ubiquitous in most of the queried root samples and in some of the soil samples (Fig. 7A). Only clone B893 showed a significantly higher abundance in the root samples than in the soil samples (*P* < 0.05 by a Wilcoxon test) (Fig. S5). B893 was found in 16/20 of the root-associated data sets that we examined (compared with 8/20 soil data sets) (Fig. 7B). In contrast, none of the five BGCs were found in any of the gut microbiome communities analyzed, and very few were detected in the aquatic environments (Fig. 7B).

10.1128/mSystems.00866-20.5 FIG S5 Relative abundances of recovered gene clusters in root-associated and soil metagenomic samples. Abundance was normalized to the *gyrB* and *rpoB* housekeeping genes within each data set (*n* = 20 per environment). Statistical differences were calculated using a Wilcoxon nonparametric test. *, *P* < 0.05; NS, not significant. Download FIG S5, TIF file, 0.2 MB. Copyright © 2020 Dror et al. 2020 Dror et al. This content is distributed under the terms of the Creative Commons Attribution 4.0 International license.

## DISCUSSION

NRPs and PKs produced by root-associated microbial communities play an important role in plant root ecosystems (38, 39). Several studies have identified and characterized BGCs and/or associated metabolites in prominent plant-growth-promoting and biocontrol agents originally isolated from plant roots (40, 41). However, the large fraction of uncultivated bacteria in root ecosystems and the limitations associated with culturing bacteria encouraged us to examine the composition of genes encoding NRPS and PKS in root environments using culture-independent approaches. These methods have been applied previously to understand SM-encoding gene diversity and distribution in bulk soil (17, 26, 42, 43), but NRPSs and PKSs have not been comprehensively explored in root ecosystems. Furthermore, to the best of our knowledge, this is the first study to explore the expression of secondary-metabolite-associated genes in root ecosystems.

Our results demonstrate distinct compositions of NRPSs and PKSs in plant (tomato and lettuce) roots relative to adjacent bulk soil, that these genes in root microbial communities are less diverse than those found in soil microbiomes, and that a fraction of these NRPSs and PKSs are highly expressed in root ecosystems. This is consistent with several previous studies showing that the phylogeny and functionality of root-associated microbial communities are significantly different from those in adjacent soil (44–46). It is well established that plants interact with soil bacterial communities through the secretion of root exudates (47, 48), resulting in the selection of specific microbial populations from the soil microbiome. This appears to be the case for the recruitment of plant-growth-promoting rhizobacteria (PGPR), which are known to harbor specific SM-encoding genes (4, 49, 50). Thus, while at this point, we cannot infer the actual function of the highly abundant and expressed NRPSs and PKSs in the root environment, we can infer that they likely play a role in various processes, e.g., competition and root colonization (51, 52).

While most of the detected SM-associated genes were too novel to link to any known metabolites, a few of the highly abundant, expressed, and/or root-enriched NRPSs and PKSs were associated with known metabolites. Azalomycin F, for instance, found to be associated with sequences in both of the plant microbiome samples, is a polyketide with reported antifungal activity against a variety of phytopathogens, which is produced by different soil- and root-associated *Streptomyces* isolates (53, 54). Diaphorin, associated with sequences found only in the tomato root microbiome, is a pederin analogue known to be produced by the psyllid Diaphorina citri endosymbiont “*Candidatus* Profftella armatura” (*Betaproteobacteria*), with potential antifungal and cytotoxic activity (55). In this regard, the described culture-independent approaches are a promising platform for identifying novel BGCs and elucidating their roles in soil ecosystems and within the framework of drug discovery efforts, despite their current limitations (16, 17).

A large fraction of the highly abundant and highly expressed NRPSs and PKSs identified in this study were not assigned, or had low identity (50 to 70%), to previously characterized genes in the MIBiG repository. Recently, a previously unidentified hybrid NRPS/PKS gene cluster was found to be essential for *Rhizoctonia* suppression by an endophyte *Flavobacterium* sp. (56), highlighting the vast amount of root-associated SMs with unidentified functional roles, which undoubtedly play a pivotal role in bacterium-plant interactions.

We screened a large set of soil cosmid libraries with candidate sequences from our amplicon sequencing and metagenomic analyses that were enriched (relative to bulk soil) and/or highly abundant and expressed in tomato and lettuce roots, taking advantage of a unique culture-independent platform capable of extracting and analyzing long NRPSs and PKSs (27, 57). Five clones containing uncharacterized gene clusters with no known function, including two that were not associated with any known taxonomic group, were identified. The fact that all of these BGCs were rather common in various root-associated environmental metagenomes but rare or completely absent in other environments suggests their potential importance in these habitats. While we can only speculate as to their synthesized metabolites’ actual activity, they were associated with bacterial groups well known for their antimicrobial capacity. *Saccharothrix saharensis* (*Actinobacteria*), for instance, which contains a BGC closely related to clone B893, is a soil-dwelling bacterium known to produce an array of antimicrobials (58). This BGC likely encodes a polyene substructure, often seen in antifungal agents, as it is capable of directly disrupting the fungal membrane (59). Of particular interest is clone B481, associated with SM-associated genes from *Streptomyces cyaneogriseus*, known for its ability to produce the biopesticide nemadectin (60). We speculate that this BGC may be associated with bacterium-fungus competition in the root ecosystems.

At the broader level, our results emphasize the need to look beyond basic descriptive diversity and composition information regarding the SM capacity of microbial communities. The pipeline adopted in this study, where potentially important NRPSs and PKSs are first identified, followed by the extraction of BGCs from cosmid libraries in order to identify potentially novel BGCs, has the advantage of being resource-efficient while spawning deeper knowledge regarding potentially important gene clusters. Future studies will focus on expressing these cosmid library BGCs in suitable hosts, enabling us to characterize their encoded metabolites and test their *in vitro* and *in planta* activities against various phytopathogens. Our results coincide with studies conducted in other plants, showcasing the as-yet-unexplored diversity of NRPSs and PKSs (43, 61).

Overall, this study indicates that the root microbiome harbors a unique, diverse, and potentially novel array of SM-synthesizing genes, which are significantly different from those in the bulk soil microbiome. To enhance our current understanding, future research should focus on identifying additional factors shaping the occurrence and expression of SMs in the root microbiome (e.g., plant health, the presence of phytopathogens, and plant growth). This will undoubtedly help expose the ecological role of SMs in root ecosystems and provide a platform for drug discovery and novel and environmentally sustainable compounds for plant protection.

## MATERIALS AND METHODS

Figure S6 in the supplemental material presents a conceptual description of the pipeline applied in this study.

10.1128/mSystems.00866-20.6 FIG S6 Study conceptual pipeline scheme. Download FIG S6, TIF file, 0.4 MB. Copyright © 2020 Dror et al. 2020 Dror et al. This content is distributed under the terms of the Creative Commons Attribution 4.0 International license.

### Amplicon sequencing, shotgun metagenomics, and metatranscriptomics analyses.

Tomato soil and root samples and lettuce root samples were collected as previously described (62). Briefly, tomato (Solanum lycopersicum Heinz 4107) and lettuce (Lactuca sativa [romaine] Assaph) seedlings were planted and grown for 42 days in a random-block lysimeter experiment at the Lachish agricultural research station in Kiryat-Gat, Israel. Each sample type (soil, tomato roots, and lettuce roots) was analyzed using triplicate samples from three different lysimeters (thus, *n* = 3 for each sample type). Each triplicate consisted of a composite sample collected from 2 to 4 plants or 3 soil samples, taken from the distant edges of the lysimeters and away from plant roots. As the same soil was used throughout the experiment, and given that soil samples were collected at a sufficient distance from growing plants, bulk soil from the tomato lysimeters served as a reference point for both tomato and lettuce soils. A previous study showed that they harbored almost identical microbial communities (32). Soil samples were collected, frozen in liquid nitrogen on-site, and stored at −80°C until further analysis. Root samples were collected intact, and soil particles were removed by shaking and briefly rinsing. The roots were then lightly dried, immediately frozen in liquid nitrogen on-site, and kept at −80°C until processed. In this study, extracted DNA was used as the template for NRPS and PKS PCR amplification using degenerate primers (A3F/A7R for AD-NRPS and degKS2F/degKS2R for KS-PKS domains, as previously reported [23]). The resulting barcoded libraries were pooled and sequenced on an Illumina MiSeq instrument, employing V2 chemistry, at the University of Illinois—Chicago Sequencing Core (UICSQC). A total of 18 samples were sequenced, which included sampling location (tomato soil versus tomato and lettuce roots) and SM family (AD and KS domains), with three replicates for each treatment. The resulting sequences were processed and demultiplexed using the QIIME2 pipeline and the integrated DADA2 method (63). Exact sequence variants (ESVs) represented by fewer than 3 sequences were removed from downstream analyses. Raw amplicon sequences are available via the MG-RAST data repository under accession number mgm4862150.3. In addition, shotgun metagenome and metatranscriptome data sets of lettuce and tomato roots (*n* = 3 for each data set type [hence, 6 for tomato and 6 for lettuce]) previously generated and analyzed from the same samples were also used for NRPS and PKS identification as described below (32). Shotgun sequence data are available via the NCBI Sequence Read Archive (SRA) data repository under project accession number PRJNA602301.

### Identification and annotation of NRPSs and PKSs.

For chemical diversity analysis of NRPS and PKS gene clusters, the different data sets (ESVs generated via amplicon sequencing and metagenome/metatranscriptome-assembled genes) were aligned against the Minimum Information about a Biosynthetic Gene Cluster (MIBiG) repository (version 6 August 2018). Only core NRPS and PKS genes (AD and KS domains) were included in the analysis. Alignment was performed using the diamond blastx command line, with >50% amino acid sequence identity. To associate each NRPS or PKS hit with its potentially synthesized metabolite, an E value of <10^−40^ was used as a cutoff. Hits that did not pass this threshold were regarded as “unknown.”

For taxonomic annotation, sequences were aligned against the nonredundant (nr) BLAST NCBI database, followed by lowest-common-ancestor (LCA) classification using the MEGAN 6.15 Ultimate edition by taking the top 10% of hits and filtering for a minimum score of 50 and a maximum E value of 0.01 (64). Conversion of gene identifiers to taxonomic path was done using the mapping files provided by MEGAN as of October 2016.

### Soil library amplicon generation screening.

Metagenomic libraries were constructed as previously reported (65). Briefly, in each library, crude environmental DNA (eDNA) was extracted directly from field-collected soil, gel purified, blunt ended, ligated into cosmid pWEB::TNC (Epicenter), packaged into lambda phage, and transferred into E. coli EC100 (Lucigen). Each library was expanded to contain 20 × 10^6^ unique cosmid clones with ∼30- to 45-kb eDNA inserts and then arrayed into 768 subpools (two 384-well plates) containing ∼25,000 unique cosmid clones per well. Each subpool was then stored as a glycerol stock for the clone recovery of interesting hits and as cosmid DNA to facilitate PCR-based screening. To generate an amplicon sequence database of NRPSs and PKSs, the following two sets of degenerate primers (AD and KS) were applied to amplify the conserved regions in the adenylation and ketosynthase domains in the biosynthetic gene cluster: AD forward primer 5′-SATBTAYACSTCVGGHWCSAC-3′ and reverse primer 5′-CCANRTCNCCBGTSYKGTACA-3′, and KS forward primer 5′-TGYTCSDSSTCGCTSGTSGCS-3′ and reverse primer 5′-GTNCCSGTSCCRTGBGCYTCS-3′. The 5′ ends of the primers were augmented with MiSeq sequencing adapters followed by unique 8-bp barcode sequences identifying the soil metagenome from which they were amplified. Amplicons were pooled as collections of 96 samples and cleaned using magnetic beads. Cleaned, pooled amplicons were used as the template in a second PCR. Prior to sequencing, all PCR amplicons were quantified by gel electrophoresis and mixed in an equal molar ratio. The resulting pool was fluorometrically quantified with HS D1000 ScreenTape and sequenced on an Illumina MiSeq instrument. Reads were debarcoded and trimmed to 240 bp. The reads from each sample were clustered using UCLUST (66) to generate the 95% tags.

### Recovery of BGC clones from metagenomic library pools.

The library well locations for target AD or KS domains were identified using well-specific barcodes incorporated into the degenerate primers (27). Specific primers with melting temperature (*T_m_*) values of ∼60°C (18 to 20 bp) were designed to amplify each unique conserved sequence of interest. To recover the single cosmid clone from each library subpool, a serial dilution of the whole-cell PCR strategy was used (17). Briefly, library glycerol stocks that contained target hits from eSNaPD analysis were inoculated into 3 ml LB broth (kanamycin and chloramphenicol) and grown overnight at 37°C to confluence. The cells cultured overnight were diluted to 2,000 CFU ml^−1^, calculated by the optical density at 600 nm (OD_600_). The 384-well plates were inoculated with 50 μl of the resulting diluent (600 CFU/well) with an Eppendorf epMotion 5075 liquid handler, grown overnight, and screened using real-time PCR with a touchdown PCR program to identify wells containing target clones. Target-positive wells were diluted to a concentration of ∼5 CFU ml^−1^, and the process was repeated to identify new wells containing target clones. Five clone pools were then plated on a solid-agar plate, and target single clones were identified by clone PCR.

### Analysis of recovered gene clusters.

Recovered single-cosmid clones were miniprepped by using a QIAprep kit and sequenced using MiSeq technology. The M13-40FOR and T7 universal primers were utilized to sequence both ends of the insert sequences. Reads, amplicons, and end sequences were assembled together to generate constant contigs using Newbler 2.6 (67). Fully assembled contigs were then analyzed using an in-house annotation script consisting of open reading frame (ORF) prediction with MetaGeneMark, HMM Scan, and BLAST search. The annotation script was developed using Python and is available at the GitHub open-source repository (https://github.com/brady-lab-rockefeller/gene_annotation). Putative functions and source organisms of genes in the BGC were assigned based on the closest characterized gene in the NCBI database. KnownClusterBlast in antiSMASH 5.0 (68) was utilized to analyze the relationship between known characterized gene clusters and recovered BGCs. The structure prediction of the adenylation domains and ketosynthase domains in BGCs was given by the antiSMASH prediction, which employs three prediction algorithms, NPRSPredictor2, Stachelhaus code, and SVM (support-vector machine) prediction. These predicted building blocks were then utilized to predict a final structure combined with known characterized BGCs in cultured bacteria.

### Recovered clone search in environmental shotgun metagenomes.

AD and KS domains from all five recovered gene clusters were searched against shotgun metagenomes from four different environments: animal and human feces (gut), aquatic, soil, and root associated. We selected 20 Illumina-sequenced shotgun metagenomes from each of these ecosystems using the JGI IMG/MER advanced search option, followed by a Blastn search using the IMG website online tool. Additional filtering was performed based on an E value threshold (10^−40^) and identity (>85%). For each hit, counts were normalized using *gyrB* and *rpoB* housekeeping gene counts (obtained via the IMG/MER platform). A relative-abundance heat map was created using the pheatmap R package (69). For gene clusters with more than one AD/KS domain, results are shown only for data sets that contained all cluster-belonging domains. A gene cluster presence/absence plot was created using the ggplot2 R package. Further information regarding selected metagenome data sets is shown in Table S2.

10.1128/mSystems.00866-20.8 TABLE S2 Public shotgun metagenomes of microbial communities from four different ecosystems (animal and human gut, aquatic, soil, and root associated). Download Table S2, CSV file, 0.01 MB. Copyright © 2020 Dror et al. 2020 Dror et al. This content is distributed under the terms of the Creative Commons Attribution 4.0 International license.

### Statistical analyses.

Alpha (Simpson index) and beta (Bray-Curtis) diversity indices across environments (bulk soil versus roots and soil versus tomato versus lettuce) were calculated using the R package vegan (70). Variation in NRPS/PKS-associated genes was visualized by principal-coordinate analysis (PCoA) using the same R package. To obtain this figure, we performed ordination on an ESV count table (constructed by QIIME2) using Bray-Curtis distances, followed by plotting using the R ggplot2 package. Difference significance between groups was determined using vegan ANOSIM (analysis of similarity).

Enrichment of ESVs between soil and tomato and lettuce roots and of NRPS/PKS-related sequences between root shotgun metagenomes and metatranscriptomes was determined using DESeq2 (71). Only sequences with a corrected-adjusted *P* value of <0.1 (Wald test *P* values corrected for multiple testing by the Benjamini-Hochberg method [72]) were chosen.

### Data availability.

Raw AD and KS amplicons from the tomato root, lettuce root, and tomato bulk soil microbial communities sequenced in this study are available via the MG-RAST data repository under accession number mgm4862150.3. Previously sequenced shotgun metagenome and metatranscriptome data sets (32) are available via the NCBI SRA data repository under project accession number PRJNA602301.
