# Supplementary figures and images for: Elucidating the Diversity and Potential Function of Nonribosomal Peptide and Polyketide Biosynthetic Gene Clusters in the Root Microbiome
Source: mSystems. 2020 Dec 22;5(6):e00866-20. doi: 10.1128/mSystems.00866-20 (PMC7762793; doi:10.1128/mSystems.00866-20)

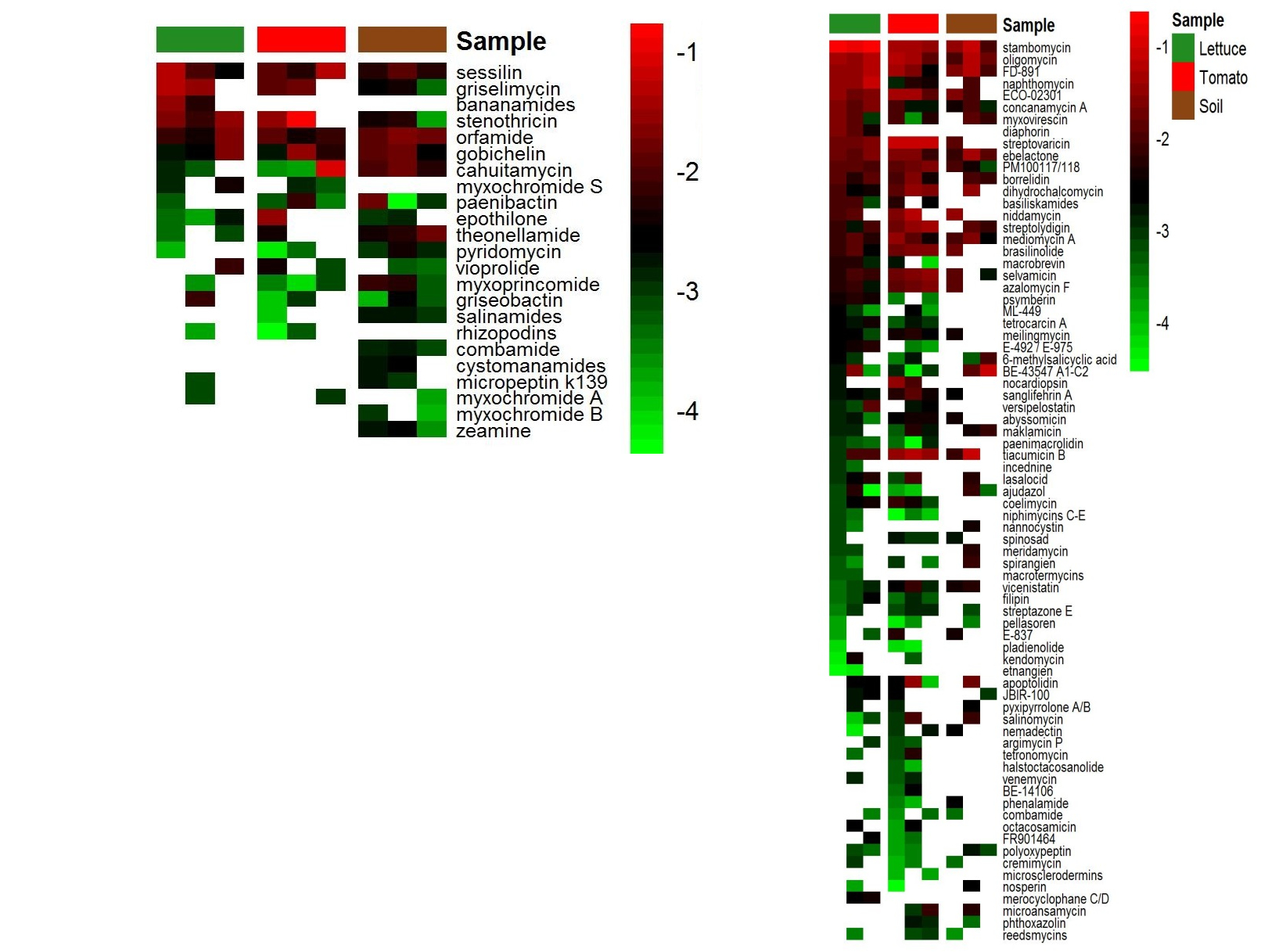

Supplement: FIG S2 [file mSystems.00866-20-sf002.tif]

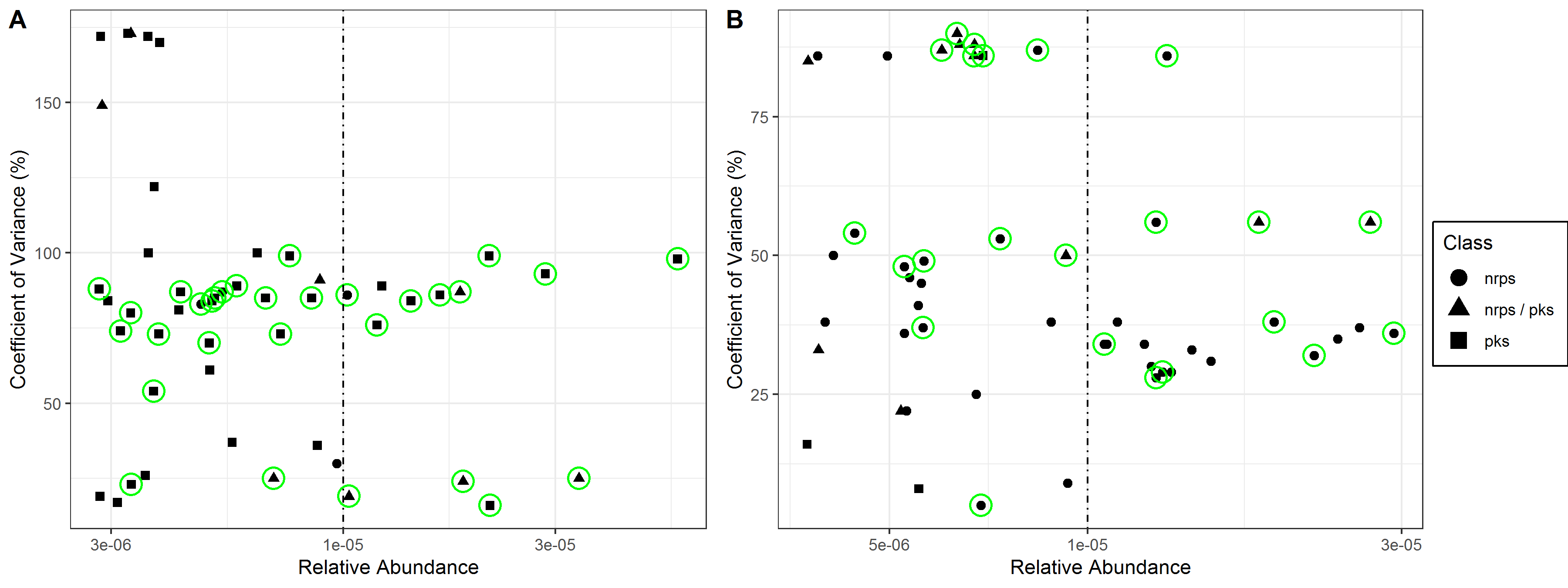

Supplement: FIG S3 [file mSystems.00866-20-sf003.tif]

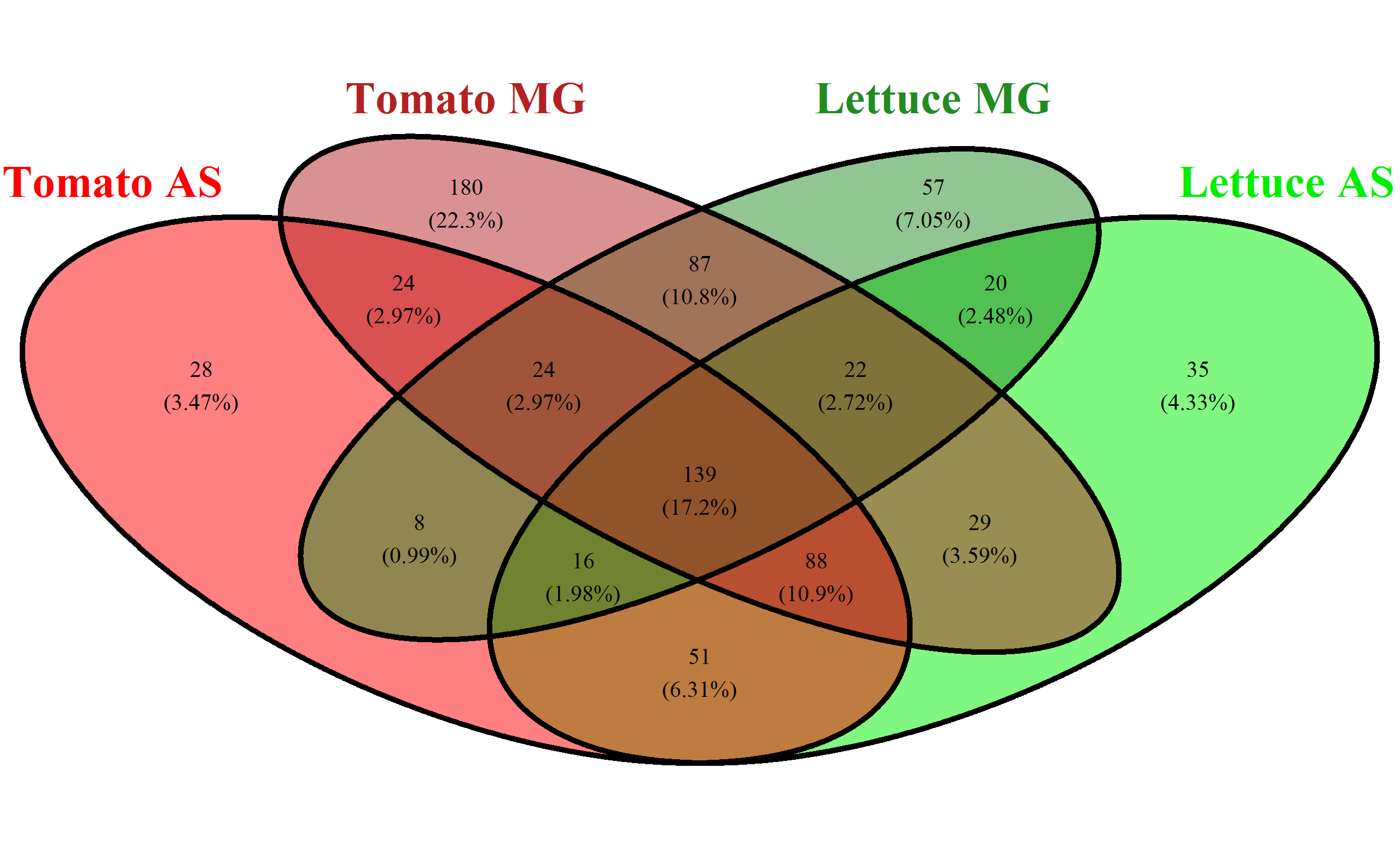

Supplement: FIG S4 [file mSystems.00866-20-sf004.tif]

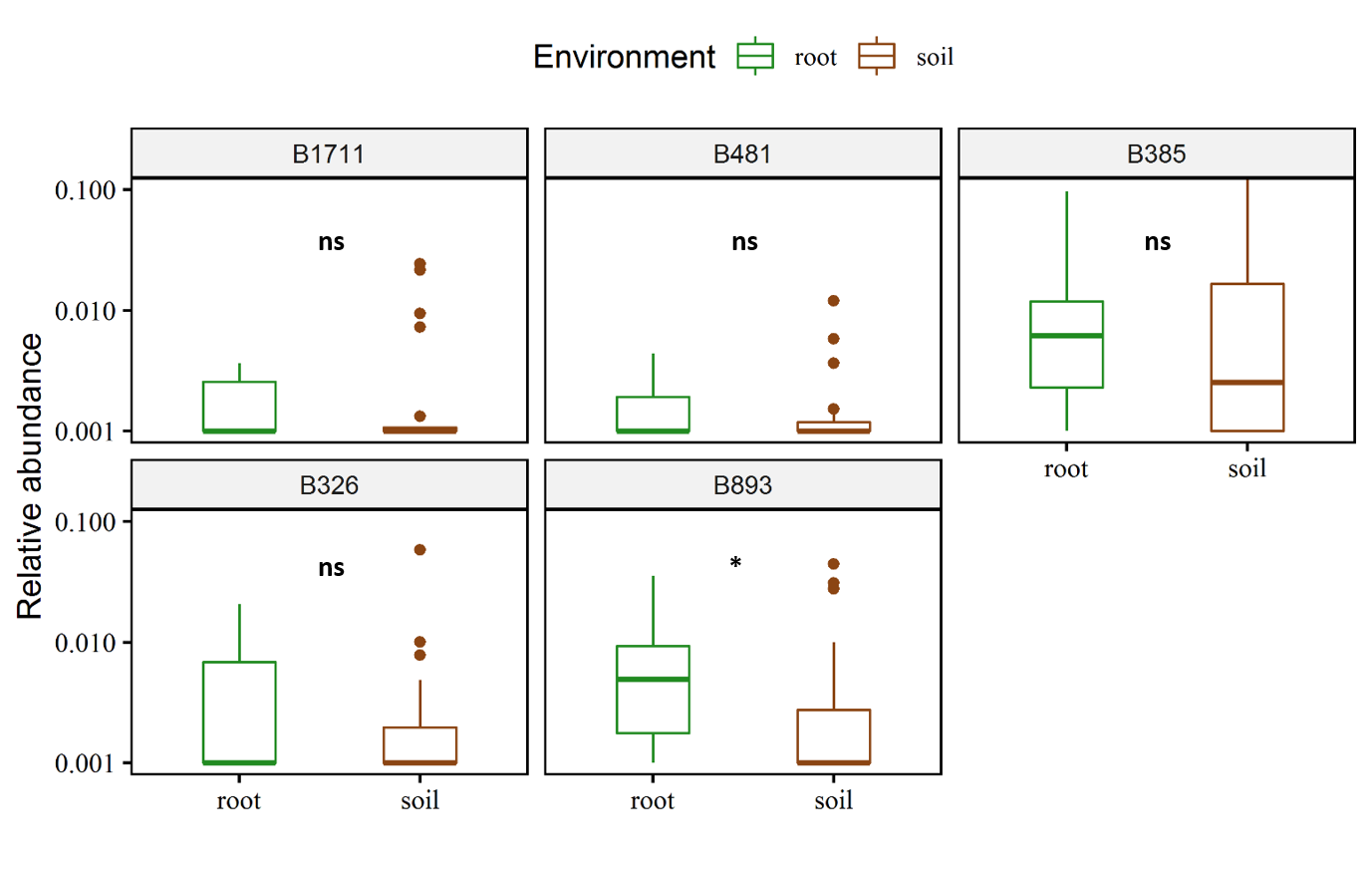

Supplement: FIG S5 [file mSystems.00866-20-sf005.tif]

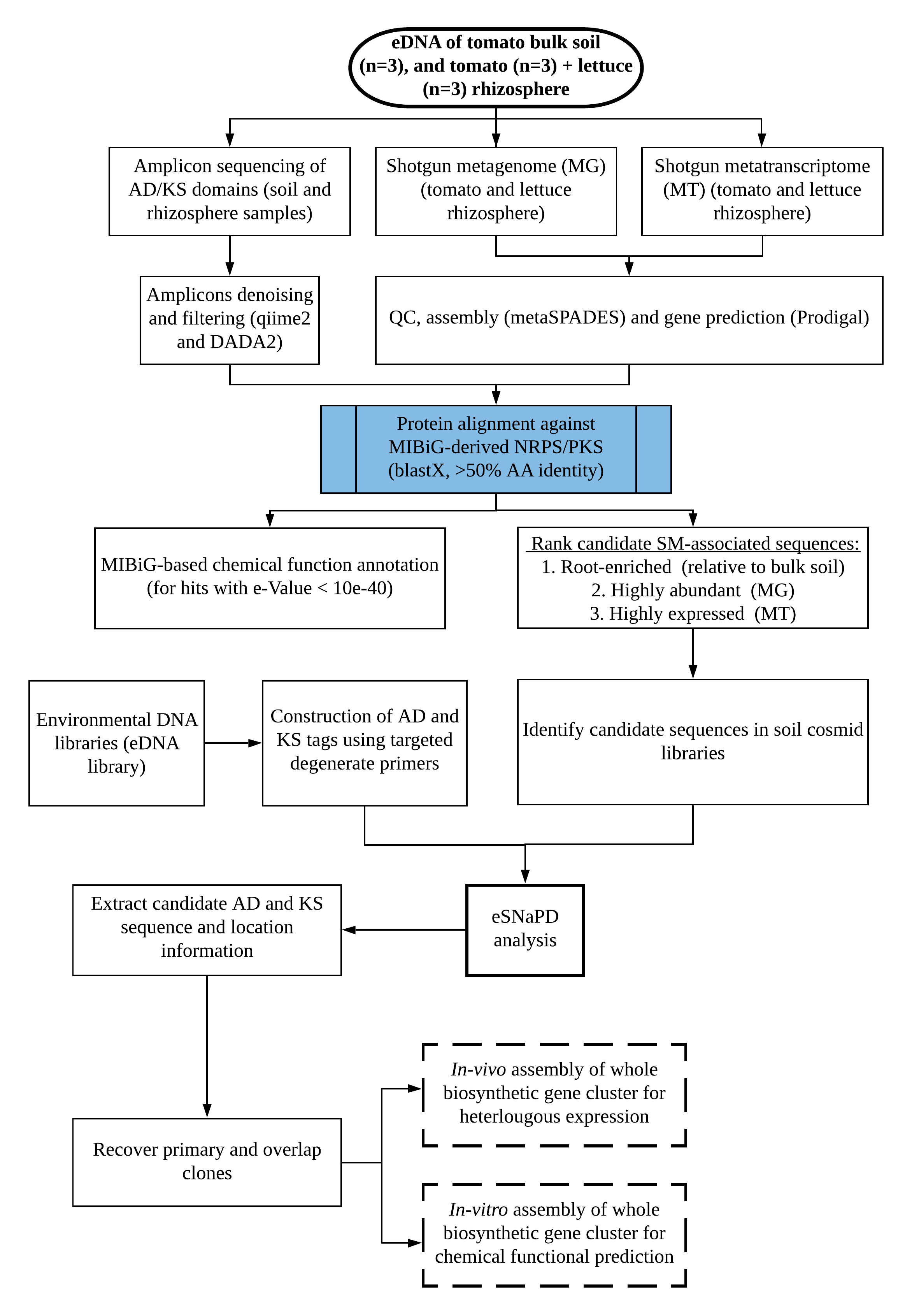

Supplement: FIG S6 [file mSystems.00866-20-sf006.tif]
